# Exogenous butyrate inhibits butyrogenic metabolism and alters virulence phenotypes in *Clostridioides difficile*

**DOI:** 10.1128/mbio.02535-23

**Published:** 2024-01-30

**Authors:** Daniel A. Pensinger, Horia A. Dobrila, David M. Stevenson, Nicole D. Hryckowian, Daniel Amador-Noguez, Andrew J. Hryckowian

**Affiliations:** 1 Department of Medicine, Division of Gastroenterology and Hepatology, University of Wisconsin School of Medicine and Public Health, Madison, Wisconsin, USA; 2 Department of Medical Microbiology & Immunology, University of Wisconsin School of Medicine and Public Health, Madison, Wisconsin, USA; 3 Microbiology Doctoral Training Program, University of Wisconsin-Madison, Madison, Wisconsin, USA; 4 Department of Bacteriology, University of Wisconsin-Madison, Madison, Wisconsin, USA; University of Oklahoma Health Sciences Center, Oklahoma City, Oklahoma, USA

**Keywords:** pathogenesis, enteric pathogens, gram-positive bacteria, metabolism

## Abstract

The gut microbiome engenders colonization resistance against the diarrheal pathogen *Clostridioides difficile,* but the molecular basis of this colonization resistance is incompletely understood. A prominent class of gut microbiome-produced metabolites important for colonization resistance against *C. difficile* is short-chain fatty acids (SCFAs). In particular, one SCFA (butyrate) decreases the fitness of *C. difficile in vitro* and is correlated with *C. difficile*-inhospitable gut environments, both in mice and in humans. Here, we demonstrate that butyrate-dependent growth inhibition in *C. difficile* occurs under conditions where *C. difficile* also produces butyrate as a metabolic end product. Furthermore, we show that exogenous butyrate is internalized into *C. difficile* cells and is incorporated into intracellular CoA pools where it is metabolized in a reverse (energetically unfavorable) direction to crotonyl-CoA and (*S*)−3-hydroxybutyryl-CoA and/or 4-hydroxybutyryl-CoA. This internalization of butyrate and reverse metabolic flow of a butyrogenic pathway(s) in *C. difficile* coincides with alterations in toxin release and sporulation. Together, this work highlights butyrate as a marker of a *C. difficile*-inhospitable environment to which *C. difficile* responds by releasing its diarrheagenic toxins and producing environmentally resistant spores necessary for transmission between hosts. These findings provide foundational data for understanding the molecular and genetic basis of how *C. difficile* growth is inhibited by butyrate and how butyrate alters *C. difficile* virulence in the face of a highly competitive and dynamic gut environment.

**IMPORTANCE:**

The gut microbiome engenders colonization resistance against the diarrheal pathogen *Clostridioides difficile,* but the molecular basis of this colonization resistance is incompletely understood, which hinders the development of novel therapeutic interventions for *C. difficile* infection (CDI). We investigated how *C. difficile* responds to butyrate, an end-product of gut microbiome community metabolism which inhibits *C. difficile* growth. We show that exogenously produced butyrate is internalized into *C. difficile*, which inhibits *C. difficile* growth by interfering with its own butyrate production. This growth inhibition coincides with increased toxin release from *C. difficile* cells and the production of environmentally resistant spores necessary for transmission between hosts. Future work to disentangle the molecular mechanisms underlying these growth and virulence phenotypes will likely lead to new strategies to restrict *C. difficile* growth in the gut and minimize its pathogenesis during CDI.

## INTRODUCTION

The Centers for Disease Control and Prevention classifies *Clostridioides difficile* as an “urgent threat” to the nation’s health, as it causes 450,000 infections, 15,000 deaths, and 1 billion dollars in excess healthcare costs per year in the United States alone (1, 2). Dysbiosis is the primary risk factor for *C. difficile* infection (CDI) and several microbial taxa directly impact *C. difficile* fitness (3–5). However, inter-individual variation in gut microbiome community composition complicates definitions of “CDIsusceptible” versus “CDI-resistant” microbiomes. Functional capacity of the distal gut microbiome (e.g., metabolites produced/consumed) differs among hosts with CDI relative to healthy hosts (6–8). These observations from human studies and animal CDI models suggest that microbiome-dependent metabolite availability, rather than microbiome composition defines CDI susceptibility and resistance. Therefore, a focus on metabolites instead of microbes may allow for more readily translatable findings.

The gastrointestinal tract contains thousands of diverse molecules derived from the diet and host/microbiome metabolism. Many of these impact *C. difficile* fitness and pathogenesis. For example, bile acids, metals, amino acids, sugars, organic acids, and short-chain fatty acids (SCFAs) affect *C. difficile in vitro* and in animal models (4–6, 9–15). SCFAs (in particular, acetate, propionate, and butyrate) are major metabolic end-products of microbiome metabolism and are primarily generated by microbial degradation of dietary fiber. Collectively, SCFAs reach concentrations >100 millimolar (mM) in bulk colon contents and are the most concentrated metabolites in the human distal gut (16). SCFA concentrations are highly dynamic and differ with respect to microbiome composition and factors like diet, antibiotic exposure, and inflammation (17).

Much of what is known about the biology of SCFAs is based on their impacts on the host. For example, SCFAs are metabolized by colonocytes or transported systemically via portal circulation. SCFAs also enhance gut barrier function and modulate immune responses. As such, SCFA deficiencies are associated with varied diseases like inflammatory bowel disease and increased susceptibility to pathogens (reviewed in reference 18). Because SCFAs are so pivotal in host-microbiome cross-talk, gut microbiome studies frequently equate a “healthy gut” with high SCFAs and a “dysbiotic gut” with low SCFAs (18). SCFAs, in particular butyrate, inhibit *C. difficile* growth and increase *C. difficile* toxin release (6, 19). Furthermore, based on data from humans and murine models of CDI, high butyrate levels are characteristic of a gut that is non-permissive to CDI (6, 15, 20). These data on CDI permissiveness, growth, and toxin release together suggest that during infection, *C. difficile* senses butyrate, perhaps as a signal of a competitive environment, and adjusts its virulence to maintain a dysbiosis-associated niche or to transmit to new hosts (18).

Despite the strong connections between butyrate and *C. difficile* fitness/pathogenesis, the molecular and genetic details of how butyrate alters growth and virulence phenotypes are poorly understood. Here, we show that exogenous butyrate induces large-scale changes in the expression of genes involved in metabolism and pathogenesis in *C. difficile*, that exogenous butyrate influences toxin release from *C. difficile* cells and the production of environmentally resistant spores necessary for transmission between hosts, that butyrate-dependent growth inhibition in *C. difficile* occurs under conditions where *de novo* butyrate synthesis pathways in *C. difficile* are active, and that exogenous butyrate is translocated into *C. difficile* cells and is metabolized through ATP- and NAD^+^-consuming pathways. Together these findings demonstrate how *C. difficile* senses and responds to butyrate and builds a strong foundation for understanding the molecular and genetic basis of these phenotypes and for exploiting these processes to improve therapeutic strategies against this pathogen.

## RESULTS

### Transcriptional profiling and virulence phenotypes of butyrate-exposed *C. difficile*

To better understand butyrate-dependent growth defects in *C. difficile* (15), we performed RNA-seq on *C. difficile* 630 grown to mid-log phase (OD_600_, ~0.3), early stationary phase (24 h post inoculation), and late stationary phase (48 h post-inoculation) in modified reinforced clostridial medium (mRCM) (15) supplemented with either 50 mM sodium butyrate or 50 mM sodium chloride. These media were pH adjusted to pH 6.5 to mimic the pH of the human distal gut (16) before experiments were performed (see Materials and Methods). The same cultures were sampled for all three time points.

**Fig 1:**
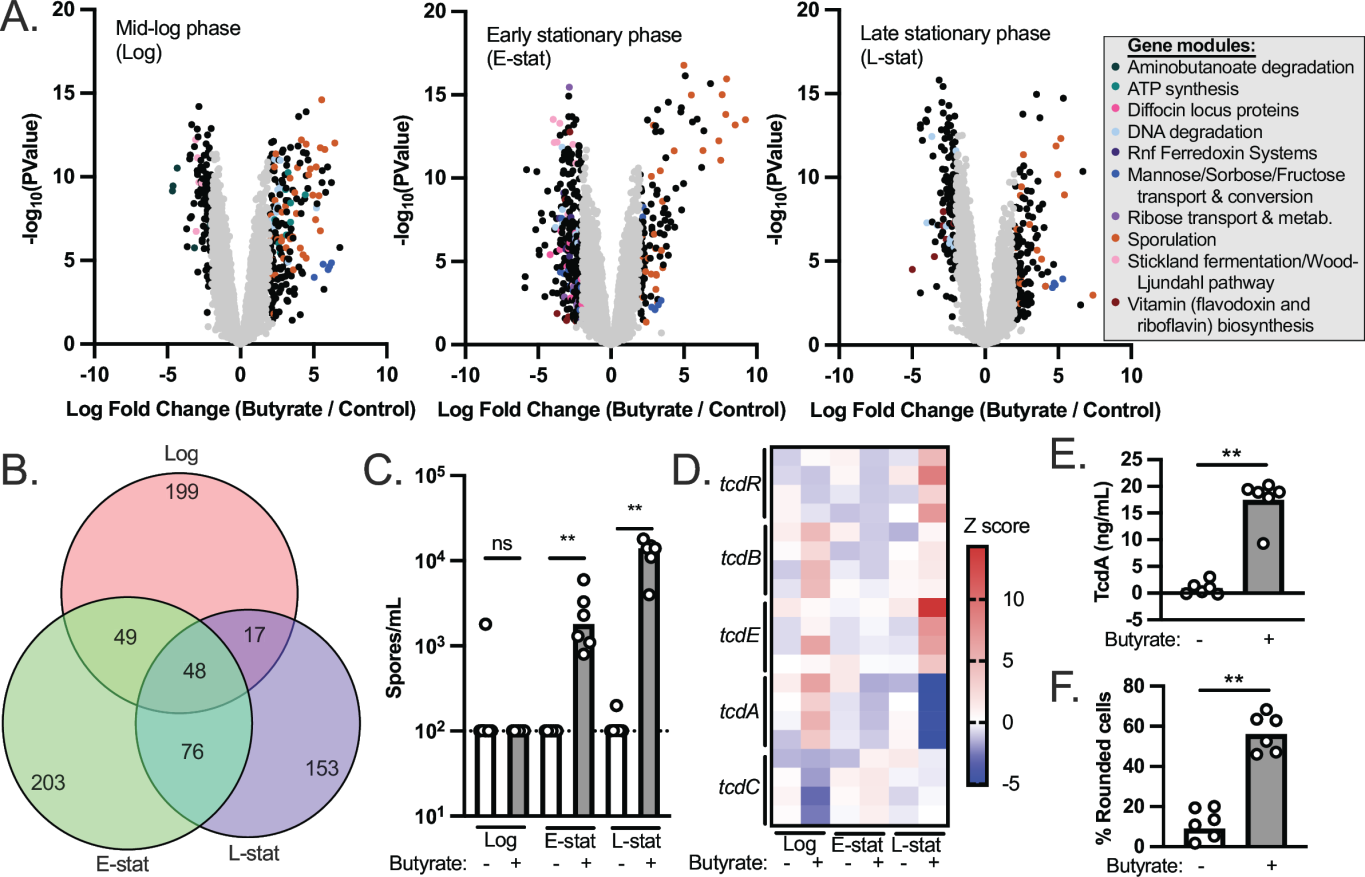
Transcriptional profiling and virulence phenotypes of butyrate-exposed *C. difficile*. (**A**) *C. difficile* 630 was grown to mid-log phase, early stationary phase, and late stationary phase in mRCM supplemented with either 50 mM sodium butyrate or 50 mM sodium chloride. RNA-seq was performed on *n* = 4 independent cultures, each of which was sampled at each growth phase. Differential gene expression between sodium butyrate (butyrate) and sodium chloride (control) are shown as volcano plots for each growth phase. Functionally related gene modules were identified among the differentially regulated genes (*P* < 0.05, log fold change > 2) using EGRIN models present in the *C. difficile* portal (21). (**B**) A Venn diagram illustrating that the majority of the significantly differentially regulated genes were unique to each growth phase. (**C**) Genes involved in sporulation were prominently differentially regulated genes in response to butyrate regardless of growth phase. We quantified spores in cultures of *C. difficile* during log phase, early stationary phase, and late stationary phase (*n* = 6 independent cultures per condition, each sampled at each growth phase). (**D**) Previous literature demonstrates that *C. difficile* toxin production is favored in the presence of butyrate (6, 19). Transcript abundance of genes in the *C. difficile* pathogenicity locus (which contains toxin genes *tcdA* and *tcdB* as well as genes involved in toxin regulation *tcdR*, *tcdE*, and *tcdC*) were compared between sodium butyrate and sodium chloride supplemented cultures from panel A. (**E**) TcdA-specific ELISA demonstrates that although *tcdA* is not upregulated at the transcriptional level, elevated levels of this toxin are present in 48 h culture supernatants of *C. difficile* exposed to 50 mM sodium butyrate relative to 50 mM NaCl (*n* = 6 independent cultures per condition). (**F**) Culture supernatants (48 h post inoculation) of *C. difficile* 630 exposed to 50 mM sodium butyrate induce more rounding of human foreskin fibroblasts relative to culture supernatants of *C. difficile* exposed to 50 mM NaCl, indicative of increased toxin activity (*n* = 6 independent cultures per condition). All bacterial growth media were adjusted to pH 6.5 prior to performing experiments. See also Tables S1–S7; and Fig. S1–S4. **P < 0.01 via Mann-Whitney test.

We observed significant differential regulation of 313, 376, and 294 genes in response to butyrate during mid-log phase, early stationary phase, and late stationary phase, respectively (Fig. 1A; Tables S1 to S3; *P* < 0.05, log fold change > |2|). To address broader functional categories of these differentially regulated genes, we used the EGRIN models present in the *C. difficile* portal (21) to group the differentially regulated genes into functionally related gene modules. Gene modules corresponding to metabolism, bacteriocin production, and sporulation were identified as being differentially expressed (Fig. 1A; Tables S4 to S6). While the majority of the differentially regulated genes were unique to each growth phase (Fig. 1B), functional enrichment of metabolic genes involved in mannose and glycine fermentation was shared across all growth phases (Table S7). One hypothesis based on these observations and based on the metabolic plasticity of *C. difficile* (8) is that butyrate alters the metabolic preferences of *C. difficile* and that these alterations manifest as differences in growth kinetics based on nutrients available in the medium. To test the hypothesis that *C. difficile* is limited for specific nutrients preferred during butyrate exposure, we supplemented mRCM with butyrate and either glycine or mannose, grew *C. difficile* 630 in these media, and monitored growth kinetics over time. The rationale behind these experiments is that by providing nutrients that are apparently preferred in the presence of butyrate, the growth rate of *C. difficile* would no longer be impaired by butyrate. Under these growth conditions, we did not observe a rescue of the butyrate-dependent growth defect (Fig. S1), suggesting that other metabolic alterations may better explain butyrate-dependent growth inhibition in *C. difficile*.

In addition to the metabolic genes noted above, we observed butyrate-dependent phenotypes relating to *C. difficile* pathogenesis. First, we noted that the most abundant class of upregulated genes, regardless of growth phase, was involved in sporulation (Fig. 1A). To validate these gene expression data, we prepared spores from *C. difficile* 630 cultures exposed to butyrate and observed significant butyrate-dependent increases in viable spores in butyrate-exposed cultures at early stationary phase and at late stationary phase (Fig. 1C). Second, previous work showed that butyrate positively impacts *C. difficile* toxin release (6, 19, 22), but we did not observe a significant increase in *tcdA* or *tcdB* transcript levels at any growth phase (Fig. 1A and D). Instead, two genes within the pathogenicity locus (*tcdE* and *tcdR*) were significantly upregulated during late stationary phase in the presence of butyrate (Fig. 1D). To situate these counterintuitive results against previously published literature, we used two independent methods (a TcdA-specific ELISA and a cell rounding assay) to confirm that, although transcription of *tcdA* and *tcdB* are not increased in response to butyrate, the extracellular levels of *C. difficile* toxins increase in response to butyrate (Fig. 1E and F). Notably, in our previous work, we showed that butyrate increases extracellular TcdB levels in *C. difficile* 630 *in vitro* in a concentration-dependent fashion (6). These data demonstrate that elevated transcripts of *tcdA* and *tcdB* are not necessary for butyrate-dependent toxin release in *C. difficile* (Fig. 1D). One possible explanation for this effect is that butyrate induces autolysis in *C. difficile* which leads to elevated levels of toxins in the extracellular space (23, 24). In support of this idea, elevated levels of lactate dehydrogenase (LDH) were detected in culture supernatants from *C. difficile* 630 grown in the presence of butyrate relative to *C. difficile* 630 grown in the absence of butyrate (Fig. S2). Importantly, we also observed butyrate-dependent changes to toxin release and sporulation (Fig. S3) and to butyrate-dependent growth in *C. difficile* R20291 (15) demonstrating that these phenotypes are not specific to *C. difficile* 630.

### Butyrate-dependent growth inhibition in *C. difficile* is associated with increased nutrient availability

**Fig 2:**
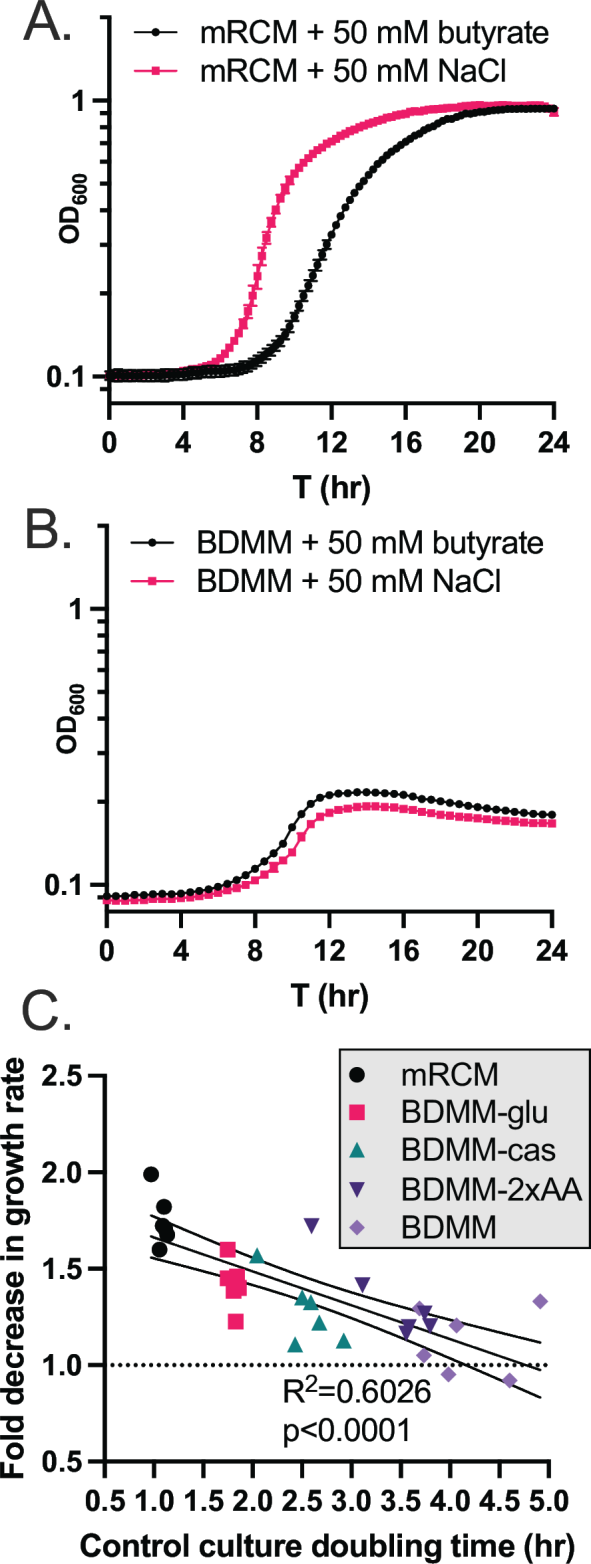
Butyrate-dependent growth inhibition in *C. difficile* as a function of growth rate. *C. difficile* 630 was grown in (**A**) mRCM and (**B**) BDMM supplemented with either 50 mM sodium butyrate or 50 mM sodium chloride (pH adjusted to pH 6.5 before performing experiments). Data points represent mean OD_600_ of three independent cultures (mRCM) and five independent cultures (BDMM) and error bars represent standard deviation. (**C**) The extent of growth inhibition by butyrate was determined for *C. difficile* grown in mRCM, BDMM, and three enriched BDMM formulations (BDMM-2xAA, BDMM-cas, BDMM-glu [see Materials and Methods]). Media were supplemented with sodium butyrate or NaCl and pH adjusted as in panels A and B. Maximum growth rates of cultures were determined using custom scripts (see Materials and Methods), and the growth rate of butyrate-supplemented cultures were compared against the growth rate of NaCl-supplemented cultures and a simple linear regression was carried out in GraphPad Prism, showing a significant correlation between growth rate in NaCl-supplemented cultures and degree of growth inhibition imposed by butyrate. The *x* axis represents the maximum growth rate of NaCl-supplemented cultures, and the *y* axis represents the fold decrease in growth rate of butyrate-supplemented cultures relative to paired NaCl-supplemented cultures. Individual data points, which represent pairs of cultures, are shown (*n* = 6 culture pairs per growth medium).

We observed that multiple metabolic pathways are differentially regulated by *C. difficile* 630 in response to butyrate (Fig. 1A and B), but we failed to rescue butyrate-dependent growth inhibition using two high-priority metabolites identified via transcriptional profiling (Fig. S1). As exemplified by the data generated on butyrate-dependent toxin release (Fig. 1D through F; Fig. S2), transcriptional profiling does not capture all relevant butyrate-dependent phenotypic changes in *C. difficile*. This prompted alternative approaches to investigate how exogenous butyrate impacts *C. difficile* metabolism. To begin this work, we grew *C. difficile* 630 in two base media: a complex rich medium (modified reinforced clostridial medium [mRCM]) (15) and a defined minimal medium (basal defined minimal medium [BDMM]) (25). These media were supplemented with either 50 mM sodium butyrate or 50 mM sodium chloride and were pH adjusted to pH 6.5 before performing experiments as above. As expected, we observed that butyrate increases the lag time and decreases the maximum growth rate of *C. difficile* 630 in mRCM (Fig. 2A) (15), but we did not observe these butyrate-dependent effects on *C. difficile* 630 growth in BDMM (Fig. 2B).

One hypothesis relating to these observations is that greater metabolic flux in *C. difficile*, regardless of specific nutrient sources present, is necessary for butyrate-dependent growth inhibition to occur. *C. difficile* can utilize a wide range of growth substrates and adapts its metabolism to fill a wide range of nutrient niches (26–28). With this metabolic plasticity in mind, we addressed this hypothesis by performing growth curve analysis of *C. difficile* 630 in three enriched BDMM formulations: BDMM +0.5% (wt/vol) glucose (BDMM-glu), BDMM +1.06% (wt/vol) amino acids (double the concentration found in BDMM [BDMM-2×-AA]), and BDMM +2% casamino acids in addition to the 0.53% amino acids found in BDMM (BDMM-cas). These media were supplemented with either 50 mM sodium butyrate or 50 mM sodium chloride and were pH adjusted to pH 6.5 before performing experiments as above. We found that growth of *C. difficile* 630 in these three BDMM variants was sensitive to the inhibitory effects of butyrate and that these effects significantly correlated with the increased growth rate observed in these media relative to BDMM (Fig. 2C; *R*^2^ = 0.6026, *P* < 0.0001). These data demonstrate that the butyrate-dependent growth defect observed *in vitro* in *C. difficile* 630 occurs as a function of general nutrient availability and is not tied to the availability or abundance of any one specific nutrient source.

### *C. difficile* butyrogenic metabolism is inhibited by exogenously applied butyrate

**Fig 3:**
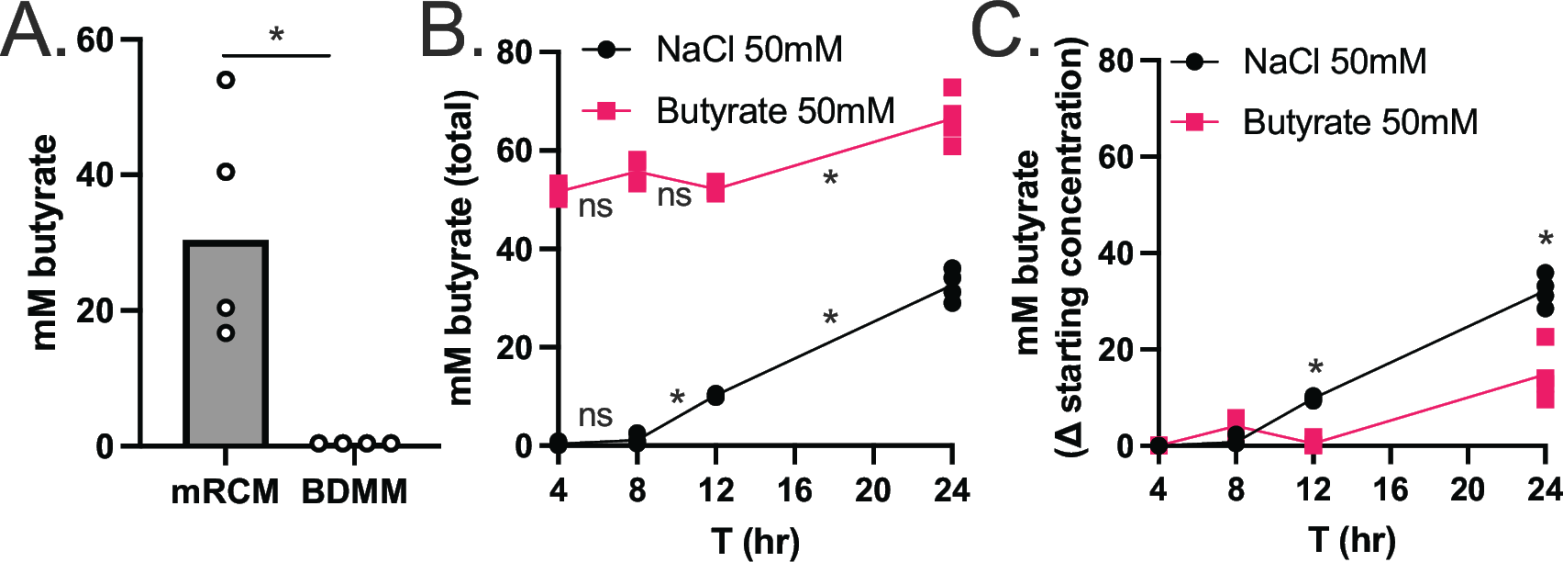
Exogenous butyrate interferes with butyrogenic metabolism in *C. difficile*. (**A**) Butyrate was quantified in 24-h culture supernatants of *C. difficile* 630 grown in mRCM and BDMM via HPLC. Individual data points represent butyrate concentration and bars represent mean concentration across *n* = 4 biological replicates per condition. (**B**) Total butyrate was quantified in culture supernatants of *C. difficile* 630 grown in mRCM supplemented with 50 mM sodium butyrate or 50 mM NaCl at 4, 8, 12, and 24 h post inoculation. (**C**) *C. difficile* butyrate production was determined for butyrate-supplemented cultures by subtracting 50 mM from the total amount of butyrate detected in panel B. All media were adjusted to pH 6.5 prior to use in experiments. For panels B and C, individual data points represent mean concentration and lines connect mean concentrations over time across *n* = 4 biological replicates per condition. Statistical significance was determined by Mann-Whitney test. *P < 0.05.

While exogenous butyrate inhibits the growth of diverse *C. difficile* strains under nutrient rich conditions (Fig. 2) (15), butyrate is a metabolic end-product of pathways in *C. difficile* that generate ATP and regenerate NAD^+^ (18). These observations led us to hypothesize that exogenous butyrate inhibits these butyrogenic pathways in *C. difficile* via product inhibition (29). To address this hypothesis, we quantified butyrate in supernatants from early stationary phase (24 h) cultures of *C. difficile* 630 grown in mRCM and BDMM. In support of our hypothesis, butyrate was detected at high abundance in mRCM supernatants but was not detectable in BDMM supernatants (Fig. 3A), and we observed a decrease in butyrate production by *C. difficile* (Fig. 3B and C) when it was grown in mRCM supplemented with butyrate. These data demonstrate that *C. difficile* does not produce butyrate as a metabolic end-product under all growth conditions and that it produces less butyrate when in a butyrate-rich environment. Furthermore, these data suggest that that decreases in butyrogenic metabolism negatively impact its growth, perhaps by decreasing metabolic flux through key energy generating and conserving pathways that are important under increasingly nutrient-rich conditions.

### Extracellular butyrate is internalized into *C. difficile*, is incorporated into CoA pools, and is metabolized in an energetically unfavorable direction

**Fig 4:**
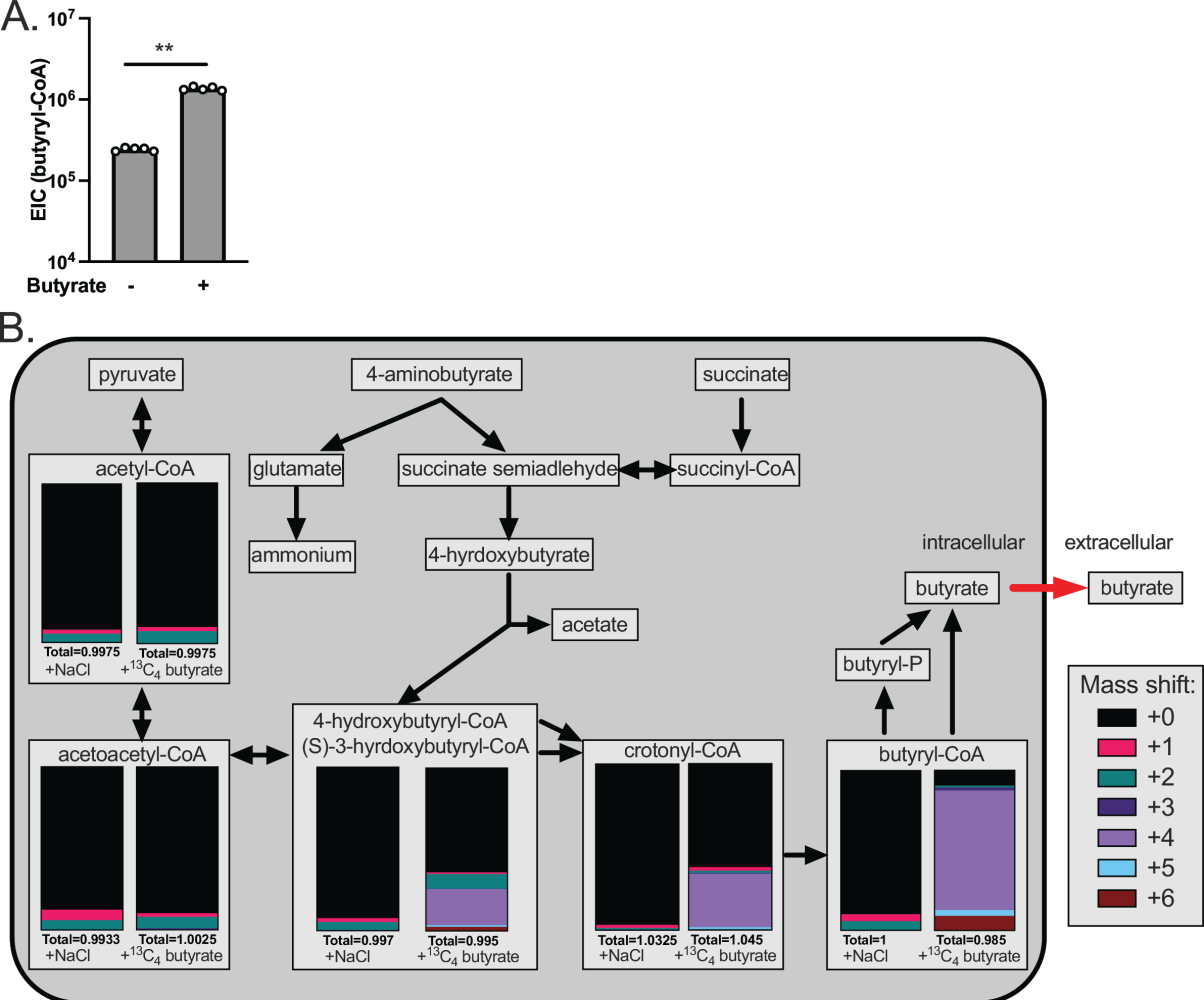
Exogenous butyrate is internalized into *C. difficile* cells, is incorporated into CoA pools, and forces energetically unfavorable metabolic processes. (**A**) *C. difficile* was grown in mRCM supplemented with 25 mM sodium butyrate or 25 mM NaCl and intracellular butyryl-CoA levels were quantified by LC/MS from cells collected at mid-log phase. Individual data points represent abundance of butyryl-CoA and bars represent mean abundance across *n* = 5 biological replicates per condition. (**B**) *C. difficile* was grown in mRCM supplemented with 25 mM ^13^C_4_ sodium butyrate or 25 mM NaCl, and isotopically labeled CoA intermediates were quantified at mid-log phase. CoA intermediates detectable by the LC/MS method under the ^13^C_4_ sodium butyrate- and NaCl-supplemented conditions are overlaid onto a metabolic map of butyrogenic pathways in *C. difficile* inferred from BioCyc (30) and arrows indicate energetically favorable directionality of the reactions. Stacked bar charts represent the mean abundance of molecules with corresponding mass shifts as annotated in the figure key (*n* = 4 biological replicates per condition). All media were adjusted to pH 6.5 prior to use in experiments. Statistical significance was determined by Mann-Whitney test. **P < 0.01.

To better understand the impacts of butyrate on *C. difficile* metabolism, we quantified intracellular pools of butyryl-CoA in *C. difficile* 630 grown in 25 mM sodium butyrate relative to *C. difficile* 630 grown in 25 mM NaCl during log-phase growth via LC/MS. We observed elevated levels of intracellular butyrate, in the form of butyryl-CoA, in butyrate-supplemented cultures relative to NaCl-supplemented cultures (Fig. 4A). These data suggest either that *C. difficile*-produced butyrate is not secreted into the extracellular environment or that exogenous butyrate enters *C. difficile* cells and is incorporated into intracellular CoA pools.

To determine whether the increase in intracellular butyryl-CoA in *C. difficile* 630 was due to elevated levels of endogenously produced butyrate that fails to be released or due to import of extracellular butyrate, we supplemented mRCM with 25 mM ^13^C_4_ sodium butyrate and examined metabolic incorporation of the ^13^C label into *C. difficile* CoA pools by LC-MS. In addition to ^13^C_4_-butyryl-CoA, we also observed increased relative abundance of ^13^C_4_-crotonyl-CoA in ^13^C_4_ sodium butyrate-exposed *C. difficile* relative to 25 mM NaCl-supplemented controls (Fig. 4B). In addition, we observed significant enrichment of molecule(s) with *m*/*z* = 853.152 containing the ^13^C_4_ label. Molecules at this *m*/*z* correspond to (*S*)-3-hydroxybutyryl-CoA and/or 4-hydroxybutyryl-CoA (Fig. 4B), which are CoA intermediates upstream of crotonyl-CoA in the butyrogenic pathways present in *C. difficile* (Fig. 4B). Importantly, (*S*)-3-hydroxybutyryl-CoA and/or 4-hydroxybutyryl-CoA are isomers and cannot be resolved with LC/MS and other CoA intermediates further upstream in these pathways were either present below the limit of detection (e.g., succinyl-CoA) or did not have ^13^C label above background (e.g., acetoacetyl-CoA & acetyl-CoA) (Fig. 4B). Regardless, the enrichment of ^13^C label in butyryl-CoA, crotonyl-CoA, and (*S*)-3-hydroxybutyryl-CoA/4-hydroxybutyryl-CoA indicates that exogenous butyrate is internalized into *C. difficile* cells and drives butyrogenic reactions in reverse (energetically unfavorable) directions in *C. difficile,* likely contributing to its reduced fitness in a butyrate-rich environment.

## DISCUSSION

This work provides several insights into how *C. difficile* responds to butyrate, a prominent gut microbiome-produced metabolite. Specifically, we demonstrate broad changes to the expression of metabolic and virulence genes, as well as increases in extracellular toxin levels and spores, in response to butyrate (Fig. 1). In addition, we demonstrate that butyrate-dependent growth inhibition in *C. difficile* occurs under nutrient-rich conditions where *C. difficile* produces butyrate from its own butyrogenic pathways (Fig. 2 and 3). Furthermore, we show that exogenous butyrate is internalized into *C. difficile* cells and is incorporated into intracellular CoA pools where it is metabolized in a reverse (energetically unfavorable) direction to crotonyl-CoA and (*S*)-3-hydroxybutyryl-CoA and/or 4-hydroxybutyryl-CoA (Fig. 4). These data support a model where *C. difficile* butyrogenic metabolism is negatively impacted by butyrate internalized from the environment and that these effects collectively lead to the butyrate-dependent growth defects and butyrate-dependent colonization resistance observed in this and previous work (6, 15). Furthermore, this work provides possible explanations for inconsistencies in previous literature, where butyrate-rich environments are not always associated with reduced *C. difficile* fitness (31, 32). Specifically, because butyrate-dependent growth inhibition occurs under conditions where *C. difficile* produces butyrate (Fig. 3A), it is possible that these previous studies did not provide the necessary environment (e.g., growth substrates and butyrate) for fitness to be negatively impacted. Continued investigation of how *C. difficile* responds to butyrate will shed light on the conditions that impact its pleiotropic effects on *C. difficile* and can serve as a model of how specific molecules, against the backdrop of a variable and complex metabolic milieu, may have differential impacts on gut resident microbes.

Beyond metabolism, the response of *C. difficile* to a nutrient- and butyrate-rich metabolic environment provides key insights into how butyrate impacts *C. difficile* virulence and highlights paths for future research. First, we observed increased toxin release by *C. difficile* in response to butyrate, as in previous literature that used both murine models of infection and *in vitro* approaches (Fig. 1E and F) (6, 19). Although a complex regulatory network controls the production of *C. difficile* toxins (33), our work highlights additional uncharacterized levels of control of butyrate-dependent toxin release by *C. difficile*. Specifically, we did not observe increases in *tcdA* or *tcdB* transcripts under any growth phase (Fig. 1A and D; Tables S1 to S3) but did observe increased levels of TcdA by ELISA and increased biologically active toxin (a combination of TcdA and TcdB) by a cell rounding assay (Fig. 1E and F). These combined observations suggest that a post-transcriptional mechanism(s) of butyrate-dependent toxin release (e.g., possibly overlapping effects from autolysis [Fig. S2] and from increased expression of the gene encoding TcdE, a holin-like protein that facilitates release of *C. difficile* toxins [Fig. 1D]) is responsible for this phenotype. Second, we observed butyrate-dependent increases in sporulation-related genes at all growth phases and in viable spores during stationary phase (Fig. 1A through C). While numerous regulators of sporulation in *C. difficile* have been characterized (34–37), the specific effects of butyrate on sporulation remain uncharacterized. Interestingly, we did not observe butyrate-dependent production of viable spores in 70:30 medium (Fig. S4), a medium formulated to induce high levels of sporulation in *C. difficile*. 70:30 medium was developed because other media (e.g., BHIS, SMC) either did not promote high levels of sporulation or inconsistently induced sporulation across *C. difficile* strains (38). Our data partially contrast a recent report that used phase contrast microscopy to show that *C. difficile* produces more endospsores when grown in 70:30 medium containing sodium butyrate relative to butyrate non-supplemented 70:30 medium (22). However, we did not quantify spores via microscopy, and this other study did not quantify viable spores. These apparently conflicting observations suggest that, while butyrate may induce *C. difficile* sporulation gene expression in 70:30 medium, these spores may not be germination proficient. Regardless, our combined observations on the differential effects of butyrate on viable spore formation in *C. difficile* grown in mRCM and 70:30 base media (Fig. 1; Fig. S4) suggest two important points. First, the conditions present in 70:30 medium are dominant to the effects of butyrate and will be a useful tool to dissect the molecular and genetic mechanisms underlying butyrate-dependent sporulation in future work. Complementarily, similar to previous work in SMC and BHIS (38), sporulation efficiency in mRCM is very low. Sporulation efficiency is increased in mRCM (Fig. 1C) and BHIS (22) by adding butyrate to the medium, which highlights the role of this molecule in modulating sporulation programs and the utility of mRCM for dissecting these phenotypes in future work. While beyond the scope of the current study, an ongoing focus of our laboratory is to understand the molecular and genetic mechanisms underlying how butyrate and other host & microbiome co-metabolites influence sporulation and toxin production and release in *C. difficile*.

The model that butyrate (which is a marker of a competitive and inhospitable gut environment) alters *C. difficile* virulence phenotypes fits with long established paradigms of diverse pathogenic bacteria enacting virulence and transmission programs under unfavorable environmental conditions (e.g., *C. difficile*, *Enterococcus*, and *Salmonella* [39–41]). In the context of our *in vitro* observations of *C. difficile* described here, we expect that the decreased fitness, increased toxin release, and sporulation to be relevant under conditions when *C. difficile* is abundant in the gut and begins to face increasingly abundant competing microbes as the microbiome recovers from dysbiosis, such as after cessation of antibiotic treatment (42) or after a change in diet that results in abundant butyrate production by the gut microbiome (15). This may simultaneously allow *C. difficile* to cause inflammation and compete with inflammation-sensitive members of the microbiome, as has been observed for pathogens like *Salmonella* (43) and facilitate transmission of spores to new hosts. In the context of a healthy microbiome where *C. difficile* is unable to colonize, we expect that the effects of growth inhibition would dominate and contribute to colonization resistance against the pathogen and facilitate spore formation.

While this work focuses on *C. difficile*, it is unlikely that similar butyrate-dependent effects are seen in all Clostridia—due to differences in both gene content and ecological niche. A recent example illustrating this idea explored butyrate sensitivity in *Bacteroides* strains and showed that *Bacteroides thetaiotaomicron* could be engineered to be susceptible to butyrate by altering the acyl-CoA thioesterase to be less efficient at disassociating butyrate from butyryl-CoA (44). In this previous work, the acyl-CoA thioesterase from *B. thetaiotaomicron* (butyrate-resistant) was replaced with the acyl-CoA thioesterase from butyrate-sensitive *Bacteroides vulgatis*. Both butyrate sensitivity and a buildup of butyryl-CoA were associated with this mutant strain. The authors of that study hypothesized that such a “backup” of butyryl-CoA had an effect of sequestering the CoA molecule. In the context of *C. difficile*, it is possible that its acyl-CoA thioesterase(s) is inefficient, and this manifests as an increase in butyryl-CoA and energetically unfavorable flux through up-stream CoA intermediates. While beyond the scope of our study, it remains to be determined if other organisms related to *C. difficile* have alleles of acyl-CoA thioesterase (or another gene[s]) that render them insensitive to the growth inhibitory effects of butyrate. Furthermore, finer-scale dissection of which specific aspect of the butyrate pathway disruption (energetically unfavorable metabolism of butyrate, lack of NADH turnover, backup of CoA pools, or other effects) are the major factor involved in butyrate-dependent growth inhibition and which of these effects serves as the molecular signal to *C. difficile* to alter the expression of genes involved in pathogenesis remain to be determined.

In summary, our work further establishes butyrate as an important marker of a “healthy” microbiome to which *C. difficile* responds by altering its metabolic and virulence programs. This view fits with an increasing appreciation of metabolites as signaling molecules influencing physiological processes outside of metabolism (45). In the case of *C. difficile,* exogenous butyrate affects the functioning of its own butyrate synthesis pathways and elicits a response from the bacterium to increase the inflammatory state of the gut and produce environmentally resistant spores necessary for transmission.

## MATERIALS AND METHODS

### Bacterial strains and culture conditions

All bacterial growth media were pre-reduced for a minimum of 24 h in an anaerobic chamber prior to use in experiments, and all bacterial growth was done under anaerobic conditions in an anaerobic chamber (Coy).

*C. difficile* 630 and *C. difficile* R20291 (46, 47) were maintained as −80°C stocks in 25% glycerol under anaerobic conditions in septum-topped vials. *C. difficile* strains were routinely cultured on CDMN agar (*Clostridioides difficile* agar with moxalactam and norfloxacin), composed of *C. difficile* agar base (Oxoid) supplemented with 7% defibrinated horse blood (HemoStat Laboratories), 32 mg/L moxalactam (Santa Cruz Biotechnology), and 12 mg/L norfloxacin (Sigma-Aldrich) in an anaerobic chamber at 37°C (Coy). After 16–24 h of growth on agar plates under anaerobic conditions, a single colony was picked into 5 mL of modified reinforced clostridial medium (mRCM, an undefined rich medium) (15), basal defined minimal medium (BDMM; a defined minimal medium containing amino acids designed to support *C. difficile* growth) (11), or 70:30 medium (an undefined rich medium formulated to induce high levels of sporulation in *C. difficile*) (38) and grown anaerobically at 37°C for 16–24 h. Single colonies were inoculated into the medium to be used in the experiments (e.g., for experiments using BDMM, colonies are picked into BDMM, allowed to grow overnight, then back-diluted into BDMM with appropriate supplements for experiments, see below).

For *in vitro* experiments examining bacterial growth, subcultures were prepared at 1:200 dilutions in mRCM or BDMM supplemented with sodium butyrate, sodium chloride, glycine, mannose, glucose, amino acids, and casamino acids as specified in the text and in the figure legends. After addition of these media supplements, the pH of the media was adjusted as specified in the figure legends. Growth curve experiments were done in sterile polystyrene 96-well tissue culture plates with low evaporation lids (Falcon). Cultures were grown anaerobically in a BioTek Epoch2 plate reader. At 15- or 30-min intervals, the plate was shaken on the “slow” setting for 1 min and the OD_600_ of the cultures was recorded using Gen5 software (version 1.11.5).

### Transcriptional profiling of *C. difficile* in response to butyrate

*C. difficile* 630 overnight cultures were back-diluted 1:50 in 20 mL mRCM in 50 mL conical tubes with 50 mM sodium butyrate or 50 mM sodium chloride adjusted to pH 6.5. Harvest was performed at OD_600_ = 0.3–0.5 for mid-log phase samples, at 24 h post inoculation (early stationary phase) or at 48 h post inoculation (late stationary phase).

At the appropriate time points, 5mL aliquots of the cultures were diluted 1:1 in chilled 1:1 ethanol:acetone to preserve RNA (48). These samples were then centrifuged for 5 min at 3,000 × *g* at room temperature, and cell pellets were frozen at −80°C. Immediately prior to RNA extraction, cell pellets were centrifuged at 4°C at 3,000 × *g* for 1 min. Residual supernatant was removed from the cell pellets, which were subsequently washed with 5 mL nuclease free PBS. Washed pellets were centrifuged at 4°C at 3,000 × *g* for 1 min, the supernatant was removed, and the resulting pellet was resuspended in 1 mL TRIzol and processed using a TRIzol Plus RNA Purification Kit (Thermo) with on-column DNase treatment according to the manufacturer’s instructions. Purified RNA was frozen at −80°C, and RNA integrity was confirmed via BioAnalyzer (Agilent) prior to proceeding.

RNA-seq on high-quality rRNA-depleted RNA extracts (12 M paired end reads per sample) and transcript level quantification, count normalization, and differential expression analysis were performed using the *C. difficile* 630 reference genome (GCF_000009205.2_ASM920v2) for sequence alignments (SeqCenter, Pittsburgh, PA, USA). See also “Data Availability.”

### *C. difficile* spore quantification

Overnight cultures of *C. difficile* grown in mRCM were back-diluted 1:200 into pre-reduced mRCM supplemented with either 50 mM sodium butyrate or 50 mM NaCl (pH adjusted to pH 6.5) and incubated anaerobically at 37°C. Overnight cultures of *C. difficile* grown in 70:30 medium were back-diluted 1:200 into pre-reduced 70:30 medium supplemented with either 50 mM sodium butyrate or 50 mM NaCl (pH adjusted to pH 7.5, the natural pH of 70:30 medium) and incubated anaerobically at 37°C. At 8, 24, and 48 h post inoculation, 0.5 mL aliquots of each culture were taken, heated to 65°C for 20 min to kill vegetative cells, and 10-fold serially diluted in pre-reduced mRCM. The serial dilutions (10 μL) were then spotted onto pre-reduced RCM plates containing 0.1% sodium taurocholate. Dilutions were also plated onto pre-reduced RCM plates without 0.1% sodium taurocholate to confirm no vegetative cells were present. Plates were incubated for 48 h at 37°C, and colonies present on RCM plates containing 0.1% sodium taurocholate were quantified. No colonies from heat-treated samples were observed on RCM plates without taurocholate.

### *C. difficile* toxin quantification

*C. difficile* toxin was quantified in 48 h culture supernatants using two assays. First, levels of TcdA were quantified relative to a standard curve of purified TcdA using the Separate Detection of *C. difficile* Toxins A and B Kit (TGCBiomics) according to the manufacturer’s instructions.

Second, a cell rounding assay was performed (modified from reference 49). Two days before cell treatment, overnight cultures of *C. difficile* grown in mRCM were back-diluted 1:200 into mRCM containing 50 mM sodium butyrate or 50 mM sodium chloride (pH adjusted to pH 6.5). Cultures were grown anaerobically at 37°C for 48 h with 50 mM butyrate or NaCl. One day before treatment, confluent human foreskin fibroblast (HFF) cells (ATCC SCRC-1041) grown in HFF medium (see below for HFF medium composition) were harvested and counted with a hemocytometer and seeded into 48-well plates at 15,000 cells per well and incubated at 37°C under 5% CO_2_ for 24 h. On the day of experiments, *C. difficile* culture supernatants were spun (3,000 × *g* for 15 min at room temperature) and were filter sterilized.

Because butyrate reduces cytotoxicity of *C. difficile* toxins on eukaryotic cells (31), we removed butyrate from culture supernatants by size exclusion filtration to minimize these effects and our interpretation of the data. Specifically, for each culture, 5 mL of filtered culture supernatant was transferred to Amicon 100 kDa MWCO filters and was centrifuged at 3,000 × *g* for 5 min at room temperature. The filter dead volume (containing molecules >100 kDa, including *C. difficile* toxins) was then washed with 5 mL of 1× PBS and was centrifuged again, as above. Then, the washed fraction containing *C. difficile* toxins was reconstituted to 5 mL with HFF medium in order to dilute this fraction back to original concentration found in culture supernatants. Concentrations of the resulting toxin-containing HFF media were prepared at undiluted, 1:50, 1:100, and 1:150 dilutions. Then, phase contrast images were acquired using Sartorius Incucyte to confirm the health and morphology of the cells prior to treatment. Then, the medium was removed from the 24-h-grown HFF cells and toxin-containing media at the dilutions noted above were added. Cells were incubated with toxin for 4 h and imaged again. Total and rounded cells were counted manually. While all dilutions of toxin-containing media showed significant differences between butyrate- and NaCl-supplemented conditions (data not shown), the 1:100 dilution of toxin-containing HFF medium was used to generate the data in Fig. 1F.

HFF medium contains the following components by volume: 88% high glucose DMEM (Thermo Scientific 11965126), 10% heat-inactivated fetal bovine serum, 1% penicillin/streptomycin (Thermo Scientific 15140122), 1% Glutamax (L-alanyl-L-glutamine) (Thermo Scientific 35050061).

### *C. difficile* lactate dehydrogenase quantification

Overnight cultures of *C. difficile* 630 grown in mRCM were back-diluted 1:200 into pre-reduced mRCM supplemented with either 50 mM sodium butyrate or 50 mM NaCl (pH adjusted to pH 6.5) and incubated anaerobically at 37°C in sterile polystyrene 96-well tissue culture plates with low evaporation lids (Falcon). At mid-log, early stationary, and late stationary phase time points, cultures were centrifuged at 3,000 × *g* for 10 min and the resulting supernatant was collected, 0.22 µm filtered, and LDH levels were immediately quantified in culture supernatants using the CyQUANT LDH Cytotoxicity Assay (Invitrogen) according to the manufacturer’s instructions. Matched cultures were lysed with Triton X-100 (0.1% final concentration) and incubated for 10 min at room temperature under anaerobic conditions. This treatment consistently decreases the number of culturable *C. difficile* 630 cells in stationary phase liquid cultures by over 99.999% (data not shown). Triton X-100 lysates were centrifuged at 3,000 × *g* for 10 min and the resulting supernatant was collected, 0.22 µm filtered, and LDH levels were immediately quantified as above. Because small but significant changes in *ldh* transcript abundance were observed in transcriptional profiling experiments (*P* < 0.05, log fold change<|2|; see Tables S1–S3), levels of LDH present culture supernatants were normalized to LDH present in matched Triton X-100 lysed culture supernatants.

### Measurement of maximum growth rate for *in vitro* growth experiments

Raw OD_600_ measurements of bacterial cultures (see “Bacterial strains and culture conditions”) were exported from Gen5 and analyzed as previously described (15). Growth rates were determined for each culture by calculating the derivative of natural log-transformed OD_600_ measurements over time. Growth rate values at each time point were then smoothed using a moving average over 90 min intervals to minimize artifacts due to noise in OD measurement data, and these smooth growth rate values were used to determine the maximum growth rate for each culture. To mitigate any remaining issues with noise in growth rate values, all growth rate curves were also inspected manually. Specifically, in cases where the growth_curve_statistics.py script selected an artifactual maximum growth rate, the largest local maximum that did not correspond to noise was manually assigned as the maximum growth rate.

### HPLC-based quantification of butyrate in culture supernatants

Butyrate was quantified in bacterial culture supernatants as previously described (50). Overnight cultures of *C. difficile* 630 grown in mRCM and sub-cultured into mRCM with 50 mM of butyrate and NaCl bacterial cultures. At the time points specified in the figures, cultures were centrifuged at 3,000 × *g* for 5 min and the resulting supernatant was collected, 0.22 µm filtered, and stored −20˚C. Supernatants were thawed and H_2_SO_4_ was added to a final concentration of 18 mM. Samples were mixed, incubated 2 min at room temperature and centrifuged at 21,000 × *g* for 10 min at 4°C. Soluble fractions were aliquoted into HPLC vials. In addition, 100, 20, and 4 butyrate standards were prepared in mRCM or BDMM, as appliable and processed as above. HPLC analysis was performed with a ThermoFisher (Waltham, MA) Ultimate 3000 UHPLC system equipped with a UV detector (210 nm). Compounds were separated on a 250 × 4.6 mm Rezex ROA-Organic acid LC column (Phenomenex Torrance, CA) run with a flow rate of 0.3 mL min^−1^ and at a column temperature of 50°C. Separation was isocratic with a mobile phase of HPLC grade water acidified with 0.015 N H_2_SO_4_. Resulting data were analyzed with ThermoFisher Chromeleon 7 and butyrate concentrations in culture supernatants were determined by analysis against a standard curve as described above.

### LC/MS-based CoA-targeted intracellular metabolomics

Overnight cultures of *C. difficile* grown in mRCM were back-diluted 1:50 in 20 mL mRCM supplemented with 25 mM sodium butyrate, 25 mM ^13^C_4_ sodium butyrate, or 25 mM NaCl in 50 mL conical tubes and incubated 37°C. The pH of mRCM used in these experiments was at the natural pH of the media (typically 6.5–7) for the experiments using sodium butyrate and the pH of the media for the^13^C_4_ sodium butyrate experiments was adjusted to pH 6.5 as needed. Cultures harvested at mid-log phase anaerobically—5 mL of culture was deposited by vacuum filtration onto a 0.2 µm nylon membrane (47 mm diameter) in duplicate. The membrane was then placed (cells down) into 1.5 mL cold (on dry ice) extraction solvent (20:20:10 [vol/vol/vol] acetonitrile, methanol, water) in a small petri dish and swirled. After approximately 30 s, the filter was inverted (cells up) and solvent was passed over the surface of the membrane several times to maximize extraction. The cell extract was then stored at −80°C. Prior to LC/MS analysis, extracts were centrifuged at 21,000 × *g* at 4°C for 10 min. Next, ~200 µL of extract normalized to OD_600_ was dried over N_2_ gas. Extracts were resuspended in 70 µL of HPLC grade water and pelleted at 21,000 × *g* at 4°C for 10 min to remove particulates. All cultures were extracted in biological triplicate or quadruplicate.

For experiments using non-labeled sodium butyrate, extracts were analyzed by mass spectrometry as previously described except without MOPS exclusion (51). Briefly, samples were run through an ACQUITY UPLC BEH C18 column in an 18 min gradient with solvent A being 97% water, 3% methanol, 10 mM tributylamine (TBA), 9.8 mM acetic acid, pH 8.2, and solvent B being 100% methanol. The gradient was 5% solvent B for 2.5 min, gradually increased to 95% solvent B at 18 min, held at 95% solvent B until 20.5 min, returned to 5% solvent B over 0.5 min, and held at 5% solvent B for the remaining 4 min. Ions were generated by heated electrospray ionization (HESI; negative mode) and quantified by a hybrid quadrupole high-resolution mass spectrometer (Q Exactive orbitrap, Thermo Scientific). MS scans consisted of full MS scanning for 70–1,000 *m*/*z* from time 0–18 min. Metabolite peaks were identified using Metabolomics Analysis and Visualization Engine (MAVEN) (52, 53).

For experiments using ^13^C_4_ sodium butyrate, the protocol was adjusted to increase resolution of CoA conjugated molecules with the following modifications. The *m*/*z* window was adjusted to exclude <300 Daltons, the first 5 min of run was excluded, and injection volume was increased from 10 to 25 µL.

### Statistical analysis

Statistical analysis was performed using GraphPad Prism 9.1.0. Details of specific analyses, including statistical tests used, are found in applicable figure legends. *P < 0.05, **P < 0.01, ***P < 0.005, ****P < 0.001.

## Data Availability

Data on normalized transcript abundance and differential expression analysis are found in Tables S1 to S3. The raw data from the RNA-seq experiments shown in Fig. 1 and Tables S1 to S3 are available through the National Institutes of Health Gene Expression Omnibus (accession no. GSE249810).

## SUPPLEMENTAL MATERIAL

Figure S1 mbio.02535-23-s0001.pdf *C. difficile* butyrate-dependent growth defects are not rescued in mRCM supplemented with mannose or glycine.

Figure S2 mbio.02535-23-s0002.pdf Butyrate-dependent autolysis in *C. difficile* 630.

Figure S3 mbio.02535-23-s0003.pdf Butyrate-dependent sporulation and toxin release in *C. difficile* R20291.

Figure S4 mbio.02535-23-s0004.pdf Butyrate does not impact viable spore counts in *C. difficile* 630 in 70:30 medium.

Tables S6 and S7 mbio.02535-23-s0005.xlsx Gene modules enriched within the genes differentially regulated by *C. difficile* 630 in response to butyrate during late stationary phase and across all growth phases.

Table S1 mbio.02535-23-s0006.xlsx Genes differentially regulated by *C. difficile* 630 in response to butyrate during mid-log phase.

Table S2 mbio.02535-23-s0007.xlsx Genes differentially regulated by *C. difficile* 630 in response to butyrate during early stationary phase.

Table S3 mbio.02535-23-s0008.xlsx Genes differentially regulated by *C. difficile* 630 in response to butyrate during late stationary phase.

Table S4 mbio.02535-23-s0009.xlsx Gene modules enriched within the genes differentially regulated by *C. difficile* 630 in response to butyrate during mid-log phase.

Table S5 mbio.02535-23-s0010.xlsx Gene modules enriched within the genes differentially regulated by *C. difficile* 630 in response to butyrate during early stationary phase.

ASM does not own the copyrights to Supplemental Material that may be linked to, or accessed through, an article. The authors have granted ASM a non-exclusive, world-wide license to publish the Supplemental Material files. Please contact the corresponding author directly for reuse.
