# Supplementary material for: Exogenous butyrate inhibits butyrogenic metabolism and alters virulence phenotypes in Clostridioides difficile
Source: mBio. 2024 Jan 30;15(3):e02535-23. doi: 10.1128/mbio.02535-23 (PMC10936429; doi:10.1128/mbio.02535-23)
Supplement: Figure S1 — C. difficile butyrate-dependent growth defects are not rescued in mRCM supplemented with mannose or glycine. [file mbio.02535-23-s0001.pdf]

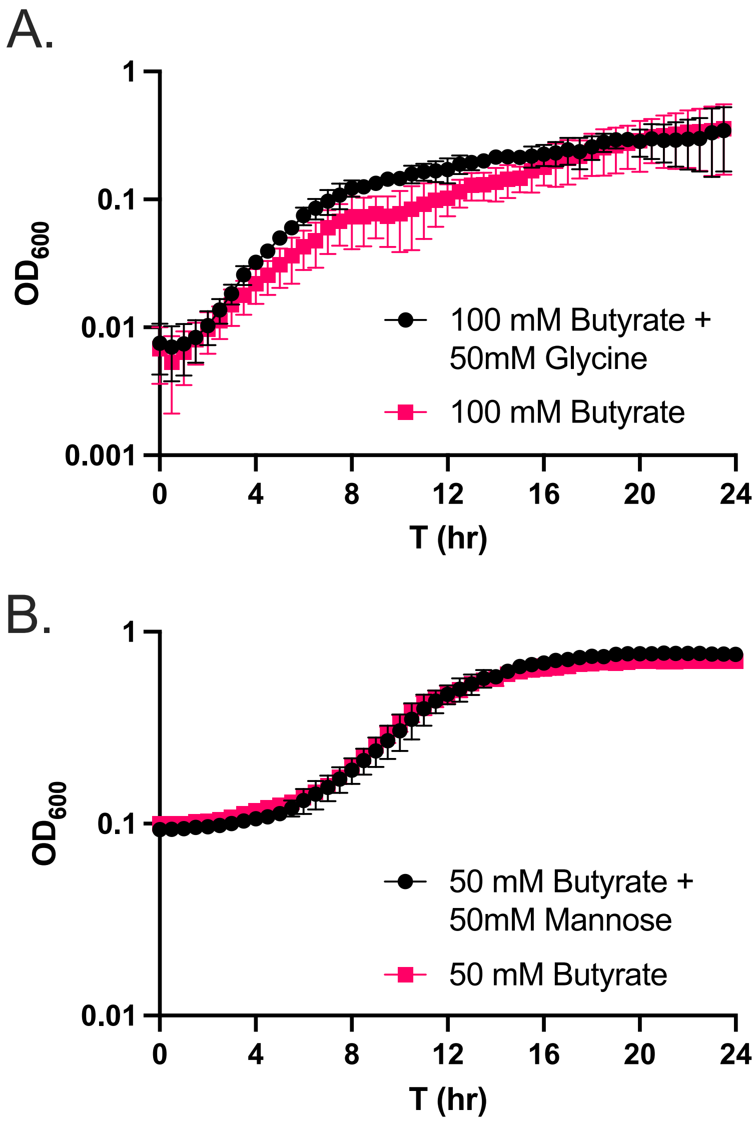

Figure S1. *C. difficile* butyrate-dependent growth defects are not rescued in mRCM supplemented with mannose or glycine. (A) *C. difficile* 630 was grown in mRCM supplemented with 100 mM sodium butyrate or with 100 mM sodium butyrate + 50 mM glycine. **(B)** *C. difficile* 630 was grown in mRCM supplemented with 50 mM sodium butyrate or with 50 mM sodium butyrate + 50 mM mannose. All media were adjusted to pH=6.5 prior to use in experiments. Data points represent mean OD_{600} of three independent cultures and error bars represent standard deviation. Related to Figure 1.
