# Supplementary material for: Exogenous butyrate inhibits butyrogenic metabolism and alters virulence phenotypes in Clostridioides difficile
Source: mBio. 2024 Jan 30;15(3):e02535-23. doi: 10.1128/mbio.02535-23 (PMC10936429; doi:10.1128/mbio.02535-23)
Supplement: Figure S2 — Butyrate-dependent autolysis in C. difficile 630. [file mbio.02535-23-s0002.pdf]

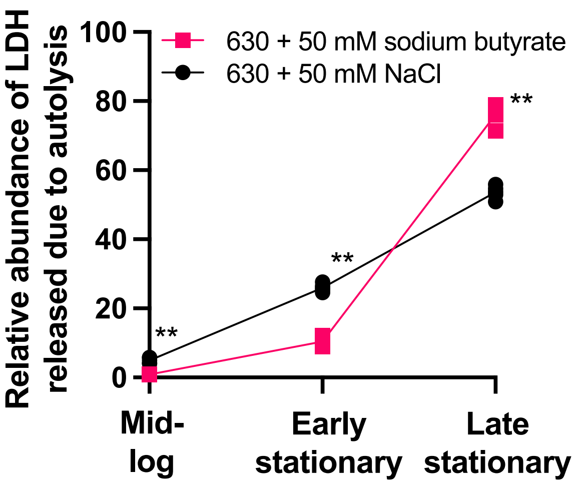


**Figure S2. Butyrate-dependent autolysis in *C. difficile* 630.** *C. difficile* 630 was grown in mRCM + 50 mM NaCl and mRCM + 50 mM sodium butyrate. Lactate dehydrogenase (LDH) was quantified in culture supernatants at mid-log phase, early stationary phase, and late stationary phase as described in Methods (n=5 independent cultures per condition). Levels of LDH detected in each culture supernatant was normalized to the average quantity of LDH detected in n=5 time point- and supplement-matched LDH Triton X-100 treated cultures to account for possible differences in LDH expression between experimental conditions (see Tables S1-S3). Individual data points represent percent LDH due to autolysis quantified in independent cultures. Lines connect mean percent LDH due to autolysis between time points. Media were adjusted to pH=6.5 prior to use in experiments. Statistical significance was determined for relevant pairwise comparisons by Mann-Whitney test. **=p<0.01.
Related to Figure 1.
