# Supplementary material for: Exogenous butyrate inhibits butyrogenic metabolism and alters virulence phenotypes in Clostridioides difficile
Source: mBio. 2024 Jan 30;15(3):e02535-23. doi: 10.1128/mbio.02535-23 (PMC10936429; doi:10.1128/mbio.02535-23)
Supplement: Figure S3 — Butyrate-dependent sporulation and toxin release in C. difficile R20291. [file mbio.02535-23-s0003.pdf]

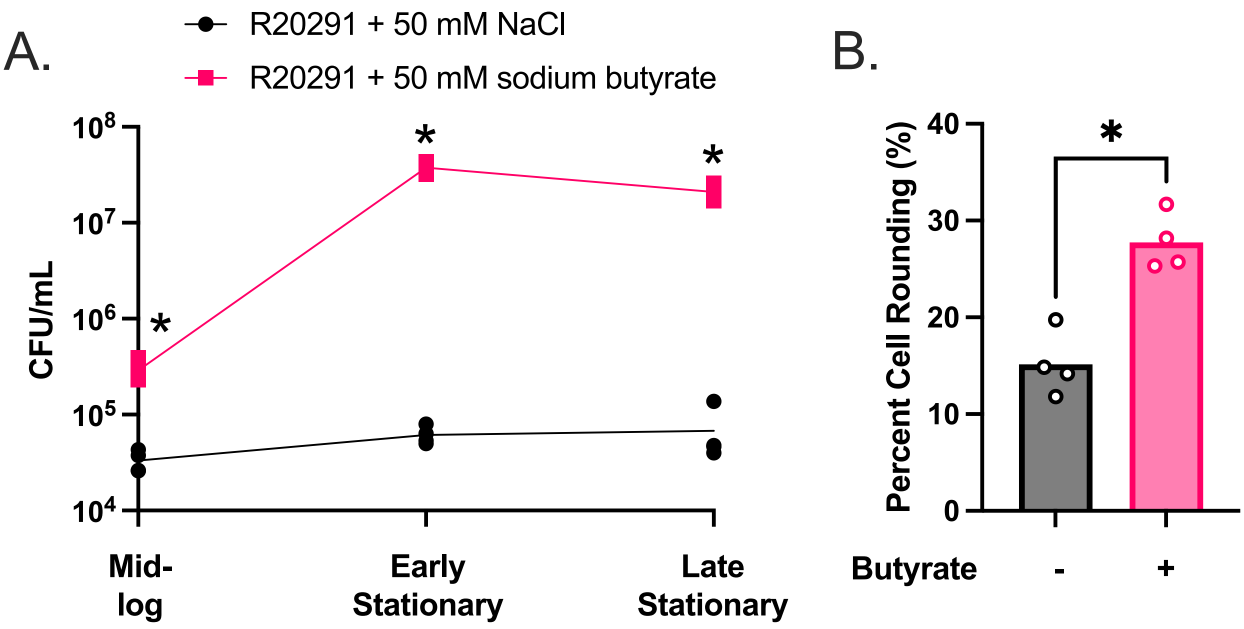

Figure S3. Butyrate-dependent sporulation and toxin release in *C. difficile* R20291.
C. difficile R20291 was grown in mRCM + 50 mM NaCl and mRCM + 50 mM sodium butyrate. **(A)** Spores were quantified at 8 hour (mid-log), 24 hour (early stationary), and 48 hour (late stationary) time points as described in Methods and **(B)** culture supernatants (48 hours post inoculation) were prepared, applied to monolayers of human foreskin fibroblasts, and rounded cells were quantified as described in Methods. Individual data points represent measurements for n=4 independent cultures per condition. Lines connect mean spore counts between time points in panel A and bars represent mean measurements at each time point in panel B. Media were adjusted to pH=6.5 prior to use in experiments. Statistical significance was determined for relevant pairwise comparisons by Mann-Whitney test. *=$p<0.05$.
Related to Figure 1.
