# Supplementary material for: Exogenous butyrate inhibits butyrogenic metabolism and alters virulence phenotypes in Clostridioides difficile
Source: mBio. 2024 Jan 30;15(3):e02535-23. doi: 10.1128/mbio.02535-23 (PMC10936429; doi:10.1128/mbio.02535-23)
Supplement: Figure S4 — Butyrate does not impact viable spore counts in C. difficile 630 in 70:30 medium. [file mbio.02535-23-s0004.pdf]

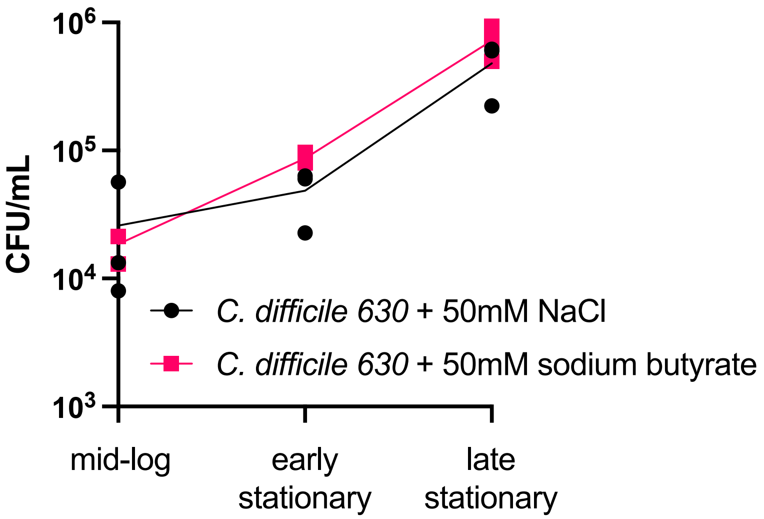

Figure S4. Butyrate does not impact viable spore counts in *C. difficile* 630 in 70:30 medium. *C. difficile* 630 was grown in 70:30 medium + 50 mM NaCl or in 70:30 medium + 50 mM sodium butyrate and spores were quantified as described in Methods. Data points represent spore counts for n=3 independent cultures per condition per time point. Lines connect mean spore counts between time points for each condition. The same cultures were sampled throughout the time course. Media were adjusted to pH=7.5 (the natural pH of 70:30 medium) prior to use in experiments. Related to Figure 1.
